# Gender inequality and adolescent suicide ideation across Africa, Asia, the South Pacific and Latin America – a cross-sectional study based on the Global School Health Survey (GSHS)

**DOI:** 10.1080/16549716.2019.1663619

**Published:** 2019-09-23

**Authors:** Rebecka Assarsson, Solveig Petersen, Björn Högberg, Mattias Strandh, Klara Johansson

**Affiliations:** a Department of Epidemiology and Global Health, Umeå University, Umeå, Sweden; b Department of Social Work, Umeå University, Umeå, Sweden; c Center for Research on Child and Adolescent Mental Health, Karlstad University, Karlstad, Sweden

**Keywords:** Gender and Health Inequality, Gender equality, adolescents, suicide ideation, global health, mental health, child, inequality, low income populations, gender, suicide

## Abstract

**Background**: Suicide ideation is a health issue affecting adolescents worldwide. There are significant variations in suicide ideation between countries and genders, which have not been fully explained. Research is especially lacking in countries outside Europe and North America. Gender equality has been shown to matter in other aspects of adolescent mental health, such as life satisfaction, but has not been researched in relation to suicide ideation at national level.

**Objective**: To investigate how national gender inequality is related to self-reported suicide ideation among adolescents, and whether this association differs between boys and girls.

**Methods**: This is a cross-national, cross-sectional study using individual survey data from the Global School-based Student Health Survey, a survey in Africa, Asia, Latin America and the South Pacific, developed and supported by among others the WHO and the CDC; connecting this to national data: the gender inequality index from the UNDP; controlling for GDP per capita and secondary school enrolment. The data was analysed using a multilevel logistic regression method and included 149,306 students from 37 countries.

**Results**: Higher national gender inequality, as measured by the gender inequality index, was significantly associated with a higher likelihood of suicide ideation in both girls and boys (odds ratio: 1.38 p-value: 0.015), but for girls and both sexes this was only after adjusting for selection bias due to secondary school enrolment (as well as GDP/capita). Interaction models showed that this association was stronger in boys than in girls.

**Conclusions**: National gender inequality seems to be associated with higher levels of suicide ideation among adolescents in mainly low- and middle-income countries, especially among boys.

## Background

Suicide is a pressing global health issue, and among youth aged 15 to 24 years it is the second leading cause of death [1]. Three out of four suicides worldwide occur in low- and middle-income countries [2], but studies on suicidality tend to focus on high-income countries mainly in North America and Europe.

Suicide is closely linked to suicide attempts and suicidal thoughts, also called suicide ideation, and together they can be referred to as suicidal thoughts and behaviours [3]. A recent meta-analysis in high-income countries, mainly US, found an odds ratio of 3.26 for future suicide attempts and an odds ratio of 32.16 to die by suicide, among adolescents with previous suicide ideation compared to those with no suicide ideation [4].

Suicide ideation among adolescents has been reported to vary considerably between different contexts and countries [4–8]. There is, however, a knowledge gap concerning the reasons for this observed international variation, especially regarding low- and middle-income countries. A comparison of survey-data from 49 countries, primarily low- and middle-income countries, indicates large cross-country variations in adolescents’ suicide ideation among 13 to 15-year-old school pupils, from less than one percent in Myanmar to more than a third of respondents in Zambia [6]. Since several risk factors for suicide pertain to various life circumstances [9], one possible explanation could be the differing life circumstances between countries.

Not only the prevalence of suicide ideation but also the gap between boys and girls differs between countries [10]. Canetto (2008) argues that these differences are in part due to diverse cultural gender norms [11]. How gender norms and gender equality are connected to suicidal behaviour is not fully understood, though it is well established that gender aspects related to suicidal behaviour are complex. Men and boys are more likely to die from suicide compared to women and girls, while, on the contrary, suicide ideation, suicide attempts, and depression are more common among women and girls [2,12–14]. A recent meta-analysis of studies covering mainly North America and Europe, except single studies from China, Brazil and Turkey, showed an odds ratio for suicide attempts of 1.96 for girls relative to boys, and of death by suicide of 2.50 for boys relative to girls [15]. However, other studies indicate that the gendered pattern of suicidal thoughts and behaviour is not consistent across countries and regions. The ratio of male/female suicides ranges from 0.9 in the western pacific region to 4.1 in the European region [2], and girls-to-boys ratio of suicide ideation among adolescents has been shown to range from 2.88 (Montserrat) to 0.75 (Tajikistan) [6].

Many attempts have been made to explain the gender complexities in suicidal behaviour [12,16–19]. Explanations often highlight gendered norms and gender relations, for example that in many contexts, it is more acceptable for women to seek help, that alcohol use and abuse (a risk factor for suicide) is more acceptable for men, and that men stereotypically use more lethal methods when attempting suicide [11,16,20,21]. Thus, the gendered norms and structures of the context in which adolescents live could potentially have an impact on both the prevalence and the gender ratio of suicidal thoughts and behaviours among adolescent boys and girls. Nowotny et al. (2015) studied US adolescents, aged 13 to 22, and found an association between being in a context with restrictive gender norms and higher likelihood of suicide ideation, which was significant for girls only, not for boys [22].

Gender equality is defined by UN Women as the equal rights, responsibilities and opportunities of women and men and girls and boys [23]. It is considered an important determinant of population health [24] including different aspects of adolescent mental health [25]. One multi-country study showed higher national gender equality to be associated with higher life-satisfaction among adolescents in Europe and North America. The association was similar for boys and girls and was explained as being due to a stronger climate of individual social support among family members, peers and classmates in more gender equal countries [25]. The results are consistent with research targeting adults [26]. However, this association has only been studied in high-income countries mainly in Europe and North America, and gender equality can be assumed to represent somewhat different aspects in low- and middle-income countries, where women’s participation in the labour force may be a sign of economic necessity rather than gender equality, and where maternal mortality is still a considerable problem. Overall, there is a lack of studies unravelling how national gender equality relates to suicide ideation, partly because suicide ideation is difficult to measure on national level.

The aim of this study is thus to investigate how national gender inequality is related to self-reported suicide ideation among adolescents, and whether this association differs between boys and girls.

As noted above, previous research on this topic has mainly been performed in North America and Europe. The current study instead focuses on countries in Africa, Asia, the South Pacific and Latin America, mainly low- and middle-income countries.

## Method

This was a cross-national, cross-sectional study analysing individual survey data in relation to national public statistics and using multilevel logistic regression.

### Ethics

This study used data that were publicly available online, and therefore, it was not relevant to apply for ethical approval.

### Data

The Global School-based Student Health Survey, GSHS, was developed by the World Health Organization (WHO) and the Centers for Disease Control and Prevention (CDC) in collaboration with the United Nations’ UNICEF, UNESCO, and UNAIDS. The GSHS is a self-reported questionnaire survey, conducted either nationally, regionally or both, in approximately 110 countries, mainly low- and middle-income countries [27]. In the surveys, students were sampled by a two-stage random cluster design. First schools were randomized in the country and then classes in the schools were randomized to be included in the study. All students in the sampled classes were invited to participate in the survey. The current study included GSHS surveys that were conducted between 2003 and 2014 on national level and included the survey question on suicide ideation. Furthermore, we only included surveys conducted in countries where there was public data available for both secondary school enrolment rates and gender inequality as measured by the Gender Inequality Index. Thirty-seven countries met these criteria (listed in Table 1). Six of these provided data from several survey years, but only data from the most recent survey year was included in the current study.

**Table 1.** A list of the 37 included countries and their characteristics.

Region	Country	Total, n	Boys, n (%)	Mean age (years)	Boys SuI (%)	Girls SuI (%)	Total SuI (%)	P-value^a^	Year of GSHS^b^	GII^c^, (data year^d^)	Log GDP/C^e^	SSE (%), (Data year^d^)
Africa	Benin	2574	1673 (65)	15.33	21.82	22.09	21.91	0.875	2009	6.21 (2010)	7.480	47.68 (2011)
	Botswana	2092	942 (45)	14.96	21.55	24.17	22.99	0.156	2005	5.23	9.241	78.53
	Ghana	2171	1134 (52)	15.32	17.37	20.73	18.98	0.046	2012	5.49 (2013)	8.216	58.36
	Kenya	3102	1514 (49)	14.44	25.69	27.46	26.6	0.267	2003	6.54 (2005)	7.480	43.14
	Malawi	2146	1006 (47)	13.97	10.44	14.04	12.35	0.011	2009	6.16 (2010)	6.871	31.84
	Mauritania	1842	862 (47)	14.96	19.26	17.24	18.19	0.264	2010	6.56	8.086	20.33
	Morocco	2380	1285 (54)	14.34	14.63	21.1	17.61	0.000	2010	5.63	8.750	62.69
	Swaziland	2533	1100 (43)	15.42	15.18	18.49	17.05	0.028	2013	5.76	9.00	62.99
	Tunisia	2344	1145 (49)	14.19	17.38	24.77	21.16	0.000	2008	3.09 (2010)	9.171	92.51
	Uganda	2938	1516 (52)	15.01	16.89	21.31	19.03	0.002	2003	5.92 (2005)	6.876	19.37
Americas	Argentina	26,430	12,546 (47)	14.57	11.94	24.09	18.32	0.000	2012	3.66	9.882	105.19
	Bahamas	1062	493 (46)	13.74	14.2	23.02	18.93	0.000	2013	3.72	10.059	92.63 (2010)
	Belize	1564	748 (48)	14.47	11.36	20.83	16.3	0.000	2011	4.11	8.969	76.03
	Bolivia	3263	1653 (51)	14.49	13.67	23.79	18.66	0.000	2012	4.92	8.683	82.88
	Costa Rica	2585	1247 (48)	14.28	7.7	14.13	11.03	0.000	2009	3.27 (2010)	9.404	99.65
	El Salvador	1795	977 (54)	14.40	10.03	19.56	14.37	0.000	2013	4.06	8.975	81.30
	Guatemala	5135	2322 (45)	14.42	10.47	20.65	16.05	0.000	2009	5.4 (2010)	8.773	57.33
	Guyana	2273	992 (44)	14.43	15.73	28.49	22.92	0.000	2010	5.22	8.653	88.64
	Honduras	1450	709 (49)	14.27	15.09	23.35	19.31	0.000	2012	4.95	8.346	72.51
	Jamaica	1474	712 (48)	14.65	18.26	25.2	21.85	0.001	2010	4.55	8.966	92.83
	Peru	2780	1351 (49)	14.51	12.07	28.34	20.43	0.000	2010	4.06	9.186	94.70
	Suriname	1567	805 (51)	14.87	9.32	18.11	13.59	0.000	2009	5.14 (2010)	9.489	75.07
	Uruguay	3402	1567 (46)	14.45	7.28	16.4	12.2	0.000	2012	3.2	9.843	107.97
Asia	Indonesia	2924	1401 (48)	13.87	3.93	5.32	4.65	0.074	2007	5.33 (2005)	8.856	70.56
	Jordan	2029	1113 (55)	14.79	16.26	18.89	17.45	0.121	2007	5.57 (2005)	9.099	93.08
	Kuwait	2586	1305 (50)	14.39	17.93	21.16	19.53	0.039	2011	2.34	11.258	93.31
	Lebanon	3890	1812 (47)	14.20	12.47	18	15.42	0.000	2011	3.98	9.661	76.13
	Malaysia	24,817	12,325 (50)	14.91	6.03	8.51	7.28	0.000	2012	3.03	10.044	69.61
	Mongolia	4471	2097 (47)	14.76	17.36	28.14	23.08	0.000	2013	2.92	9.314	91.46 (2015)
	Myanmar	2531	1275 (50)	14.51	0.71	1.04	0.87	0.372	2007	4.59 (2010)	7.892	46.26
	Pakistan	5026	3770 (75)	14.27	7.48	7.01	7.36	0.578	2009	5.7 (2010)	8.335	35.26
	Philippines	4924	2122 (43)	14.61	12.02	20.38	16.77	0.000	2011	4.49 (2010)	8.649	88.39 (2013)
	Sri Lanka	2275	1019 (45)	13.91	10.89	9.39	10.07	0.238	2008	4.09 (2010)	8.918	96.93 (2010)
	Tajikistan	9245	4628 (50)	14.60	13.46	10.05	11.76	0.000	2006	3.45 (2005)	7.403	81.40
	Thailand	2258	1130 (50)	13.92	9.2	7.54	8.37	0.152	2008	3.19 (2010)	9.414	78.22
Oceania	Fiji	1627	686 (42)	14.34	16.33	18.38	17.52	0.281	2010	3.69 (2014)	8.882	90.92 (2011)
	Samoa	1801	690 (38)	14.17	38.41	29.25	32.76	0.000	2011	4.91	8.643	86.37
		Total, n	Boys, n (%)	Mean age (years)	Boys SuI (%)	Girls SuI (%)	Total SuI (%)	P-value^a^		Mean of all countries		
Total		149,306	73,672 (49)	14.58	12.03	17.92	15.01	0.000		4.60	8.832	74.11

SuI: Suicide Ideation. GII: Gender Inequality Index. SSE: Secondary School Enrolment. GSHS: Global School-based Student Health Survey.

^a^Chi^2^- test, p-value of difference in suicide ideation between genders.

^b^Year the Global School-based Student Health Survey was conducted in country.

^c^ Gender Inequality Index. Multiplied by 10 to simplify analyzing.

^d^If different from the year of GSHS the year the data was sampled is stated in parentheses.

^e^GDP per capita is measured as PPP (purchasing power parities).

The GSHS target group was adolescents aged 13 to 17 years, but the sample included ages 11–18 years [28]. Only data from participants of ages 13–17 years were included in the current study, as the rest might not be representative of their age group, i.e. probably started school early or remained longer than usual (10,122 individuals removed). Of the remaining respondents, those who did not answer questions about gender (1,351), age (857) or suicide ideation (3,230) were also removed (3.5% of the sample within the age range). The number of students remaining in the study was 149,306.

### Measures

#### Individual variables

This study analysed a question on self-reported suicide: ‘During the past 12 months, did you ever seriously consider attempting suicide?’ with the response options yes or no. Gender and age were also self-reported. In the analysis, boys were set as the reference category, and age was included as a linear variable ranging from 13 to 17 years.

#### Macro-level indicators

As an indicator of national gender equality, the Gender Inequality Index (GII) was used. The index is compiled by the United Nations Development Program (UNDP) and includes data on reproductive health (maternal mortality rate and adolescent birth rates), gender gaps in empowerment (parliamentary seats and secondary education) and gender gaps in labour market participation [29]. The GII is publicly available online and was downloaded from the UNDP [30]. The GII ranges from zero to one, but to simplify interpretation of odds ratios, the index was multiplied by 10. A higher value indicates that the country is more unequal.

The dimensions included in the GII have been related to several aspects of gender equality which could be relevant for adolescents such as political representation, education, labour market, and reproductive health [29]. Initially, other indexes of gender inequality were also considered, i.e. the Social Institute and Gender Index (SIGI), the Global Gender Gap index (GGG) or the Gender Equality Measurement (GEM). The GEM was replaced by the GII in 2010 and therefore not an option [31]. The SIGI index was modified between 2009 and 2014, rendering the years difficult to compare [32]. The GGG is similar to the GII in many aspects but lacks maternal mortality rate and adolescent birth rate [33] and covers fewer countries, especially from earlier years. In summary, the GII was chosen because it had the highest coverage of countries, best comparability over time and included the aspects we were interested in.

GDP/capita based on purchasing power parities (PPP) was controlled for as a possible confounder. Since the relationship between income and well-being tends to be logarithmic rather than linear, GDP/capita was log-transformed using the natural logarithm [34].

Income inequality as measured by the Gini coefficients was also considered a potential confounder, but due to a lack of available data in many countries, it was not possible to control for this. The Human Development Index (HDI) by the UNDP [35] was initially controlled for but it was highly correlated (−0.784) with GII and was therefore discarded to avoid multicollinearity.

National gross secondary school participation rate was controlled for, as the survey was conducted only with students participating in school classes, and school enrolment differs substantially between the included countries. In countries with low school enrolment, we can assume that mainly adolescents from disadvantaged circumstances will be out of school, due to, e.g. costs associated with schooling, the need to start working early, or early marriage and/or pregnancy. Since disadvantaged adolescents and school drop-outs are more likely to have depression and/or suicidal thoughts or behaviours [9,36,37], the sample is likely to be biased. School enrolment is also reversely related to gender inequality: countries with higher inequality have lower school enrolment of both boys and girls (Pearsons r = −0.721 for boys’ school enrolment, −0.713 for girls’ school enrolment vs GII, n = 37 countries). Thus, in more equal countries, the at-risk adolescents are more likely to be included in the sample.

Secondary School Enrolment (SSE) was downloaded from the World Bank [38] and collected by UNESCO (United Nations Educational, Scientific, and Cultural Organization). The variable is the number of enrolled students compared to the number of children that age, which allows the rate to be above 100% if many over- or under-aged students are enrolled.

Finally, survey-year was controlled for, ranging between 2003 and 2013 (see Table 1), as a categorical variable with 2013 as reference category (coefficients not in table).

The data from the GSHS was matched to the macro-level variables according to the year of data collection. In cases when the years did not exactly match across samples, data collection years were matched as close as possible, with a maximum four years limit of differences applied.

To compensate for the study design (students clustered in schools) we used a variable for sample stratum, as a proxy for school level. Each stratum contained either two smaller schools or one larger school. (No variable for school was available in the data.)

### Analyses

Statistical analyses were performed in STATA (Version 15.1). Multilevel multivariable logistic regression was used to investigate the association between national gender equality and suicide ideation, controlling for age, sex, the year the survey was conducted, log transformed GDP/capita and gross secondary school enrolment rate. Multilevel analysis was used because this method considers the hierarchal structure of the data, as respondents clustered within strata, then clustered within countries.

Suicide ideation was modelled in relation to GII (Table 2), including interaction terms between gender and GII. Analyses stratified by sex were also performed (Table 3).

**Table 2.** The association between national gender inequality and suicide ideation among 13–17 year-olds in 37 countries. Multilevel logistic regression. (Total n = 149,306; mean [range] per country n = 4035 [1062–26,430]; 1,085 strata, mean [range] per stratum n = 138 [18–524]).

	Model 1		Model 2		Model 3		Model 4	
	OR (95% CI)	P-value	OR (95% CI)	P-value	OR (95% CI)	P-value	OR (95% CI)	P-value
Fixed effects								
GII^a,b^	1.14	0.130	1.51	**<0.001**	1.54	**<0.001**	1.64	**<0.001**
	(0.96–1.36)		(1.28–1.78)		(1.27–1.88)		(1.34–2.00)	
Age	1.11	**<0.001**	1.11	**<0.001**	1.11	**<0.001**	1.11	**<0.001**
	(1.10–1.13)		(1.10–1.13)		(1.10–1.13)		(1.10–1.13)	
Sex								
Boy^c^	1.00		1.00		1.00		1.00	
Girl	1.58	**<0.001**	1.60	**<0.001**	1.60	**<0.001**	2.45	**<0.001**
	(1.55–1.65)		(1.55–1.65)		(1.55–1.65)		(2.16–2.77)	
Sec. school enrolment rate (%)			1.02	**<0.001**	1.02	**<0.001**	1.02	**<0.001**
			(1.01–1.03)		(1.01–1.03)		(1.01–1.03)	
logGDP/Capita^d^					1.05	0.674	1.06	0.663
					(0.80–1.35)		(0.83–1.35)	
Interactions								
GII*girls							0.91	**<0.001**
							(0.88–0.93)	
Random effects								
Median Odds Ratios								
Country level	1.53		1.36		1.36		1.36	
Strata level	1.39		1.39		1.39		1.39	
Log likelihood	−59,943.7		−59,933.2		−59,933.1		−59,908.9	
Likelihood ratio test^e^	<0.001		<0.001		0.674		<0.001	

Gender Inequality Index, potential range 0–10, actual range 2.34–6.56. Higher value means less equal.

All models controlled for the year the Global School-based Health Survey was made, categorical variable (range: 2003–2013), coefficients not in table.

^c^Boys: n = 73,672 (49.34%).

^d^log GDP per capita is measured as PPP (purchasing power parities).

^e^the likelihood ratio test compares each nested model to the previous model to see if it is a significantly better fit. The first model is compared to a single-level regression.

**Table 3.** The association between national gender inequality and suicide ideation among 13–17 year-olds in 37 countries, stratified by gender.

		Model 1		Model 2		Model 3	
		OR	P-value	OR	P-value	OR	P-value
**Only girls** (Total n = 75,634; mean [range] per country = 2044 [569–13,884]; 1071 strata, mean [range] per stratum n = 71 [1–279])							
Fixed effects							
GII^a,b^		1.07 (0.90–1.28)	0.458	1.46 (1.23–1.72)	**<0.001**	1.46 (1.20–1.77)	**<0.001**
Age		1.14 (0.12–1.16)	**<0.001**	1.14 (1.12–1.16)	**<0.001**	1.14 (1.12–1.16)	**<0.001**
Sec. school enrolment rate (%)				1.02 (1.02–1.03)	**<0.001**	1.02 (1.02–1.13)	**<0.001**
logGDP/Capita^c^						1.00 (0.78–1.28)	1.000
Random effects							
Median Odds Ratio							
Country level		1.54		1.35		1.35	
Stratum level		1.41		1.41		1.41	
Log likelihood		−33,790.8		−33,778.3		−33,778.2	
Likelihood ratio test^d^		<0.001		<0.001		<0.001	
**Only boys** (Total n = 73,672; mean [range] per country = 1991.1 [493–12,546]; 1,079 strata, mean [range] per stratum n = 68.3 [1–336])							
Fixed effects							
GII^a,b^		1.25 (1.05–1.49)	**0.013**	1.56 (1.29–1.90)	**<0.001**	1.61 (1.28–2.03)	**<0.001**
Age		1.07 (1.05–1.10)	**<0.001**	1.07 (1.05–1.10)	**<0.001**	1.07 (1.05–1.10)	**<0.001**
Sec. school enrolment rate (%)				1.02 (1.01–1.03)	**<0.001**	1.02 (1.01–1.03)	**0.001**
logGDP/Capita^c^						1.07 (0.8–1.43)	0.638
Random effects							
Median Odds Ratio							
Country level		1.52		1.43		1.42	
Stratum level		1.42		1.43		1.42	
Log likelihood		−25,945.6		−25,939.6		−25,939.5	
Likelihood ratio test^d^		<0.001		**0.001**		0.6378	

^a^Gender Inequality Index, potential range 0–10, actual range 2.34–6.56. Higher value means less equal.

^b^All models controlled for the year the Global School-based Health Survey was made, categorical variable (range: 2003–2013), coefficients not in table.

^c^log GDP per capita is measured as PPP (purchasing power parities).

^d^the likelihood ratio test compares each nested model to the previous model to see if it is a significantly better fit. The first model is compared to a single-level regression.

The median odds ratio (MOR) was calculated using the STATA *xtmrho* command. The MOR is a measure of group-level variation for logistic multilevel models [39,40]. The MOR is essentially the median of all potential odds ratios between a country with lower suicide ideation and a country with higher suicide ideation, indicating the magnitude of the cross-country variation in suicide ideation.

Log likelihood ratio tests were performed, which showed if each nested model was a better fit to the data than the previous model, with the first model compared to single-level logistic regression.

Sensitivity analyses was performed as follows:

Analyses excluding outlier countries (Samoa, Kenya, Indonesia, Myanmar), with outlier defined as when residuals from the full regression model were either larger than 75th percentile plus 1.5 times the interquartile range, or smaller than 25th percentile minus 1.5 times the interquartile range.

Ecological analyses regressing national weighted, age-adjusted prevalences of suicide ideation on GII, SSE and log GDP/capita. Survey weights were available in the data to compensate for survey design and drop-out and enable generalisation to the population of *school students* in each country. (Weights were not possible with the multilevel analyses.) Regression was then weighted (analytical weights) by population size (adolescent population) of the country, and by the inverse variance (1/SE^2^) of the prevalence estimate. The latter puts emphasis on the more precise estimates [41].

## Results

### Descriptive statistics

Table 1 presents descriptive statistics for all 37 countries and all variables used in the study. The self-reported suicide ideation mean rate among adolescents was 15%. The rate, however, varied considerably between countries, ranging from 0.87% in Myanmar to 32.76% in Samoa. A significant difference in suicide ideation rates between girls and boys was found in 26 of the 37 countries. In 24 of these countries girls were more likely to report suicide ideation than boys.

National gender inequality (here measured through the Gender Inequality Index, GII, multiplied by 10) ranged from 2.34 (Kuwait) to 6.56 (Mauritania). GDP/capita in PPP ranged from 6.871 (Malawi) to 11.258 (Kuwait). Secondary school enrolment rate ranged from 19.4% (Uganda) to 107.97% (Uruguay).

### Multilevel logistic regression

Table 2 presents the results for the associations between gender inequality, measured by the gender inequality index (GII), and suicide ideation. Model 1 presents the odds ratio of suicide ideation in relation to national gender inequality, controlling for sex, age and the year of the survey. It shows no association between suicide ideation and gender inequality. When also controlling for secondary school participation rates in model 2, higher gender inequality in the country was significantly associated with higher likelihood of suicide ideation. This relationship was not affected when also controlling for log GDP/capita in model 3. Higher secondary school enrolment was also in itself associated with higher likelihood of suicide ideation in all models.

In all models, being a girl was related to higher odds of suicide ideation. The interaction term in model 4 shows that the relationship between gender inequality and suicide ideation was significantly weaker for girls than for boys. This result is clarified in Table 3, where stratified analyses by gender are presented. The association between GII and girls’ suicide ideation in the fully controlled model 3 was 1.46 (p-value <0.001), while the corresponding odds ratio for boys was 1.61 (p-value <0.001). For the boys, the association between GII and suicide ideation was significant also before controlling for secondary school enrolment, odds ratio 1.25 (p-value 0.013).

The Median Odds Ratio (MOR) for the country level, indicating the magnitude of the cross-country variation in suicide ideation, was relatively high, 1.53 in the first model, decreasing somewhat after introducing secondary school enrolment, but not changing when introducing GDP per capita or the interaction term. In a model with only age and sex, the MOR was 1.85 (data not in a table).

In sensitivity analyses excluding four outlier countries (Myanmar, Indonesia, Samoa, Kenya), the associations between national gender inequality and suicide ideation remained significant. An exception was analyses excluding *only* Myanmar (lowest suicide ideation), where associations were no longer significant; unless also excluding at least Indonesia (second lowest suicide ideation). Results were stable when excluding any of the other outliers one by one.

Ecological analyses using weighted, age-adjusted prevalences yielded similar conclusions: GII was related to suicide ideation only after controlling for secondary school enrolment.

## Discussion

This study found that higher levels of national gender inequality were associated with higher levels of adolescent suicide ideation; but for girls and the combined sample, this was only significant after controlling for secondary school enrolment. The association differed by gender, i.e. national gender inequality had a stronger relation to suicide ideation in boys compared to girls; and was for boys significant even before controlling for selection bias due to school enrolment. The lack of associations for girls and both sexes combined prior to controlling for school enrolment indicates that *in the population of school students*, there’s likely no relation between national gender inequality and suicide ideation, while such a relation might exist *in the population of adolescents in general*. The composition of the population of school students will differ considerably between countries, since school enrolment differed, and the inclusion or exclusion of students in settings with less than 100% school enrolment is not likely to be random.

Overall, suicide ideation among adolescents was found to be relatively common; and in most countries, more common in girls than in boys. While keeping in mind that high rates of suicide ideation does not automatically translate to high rates of suicide mortality, suicide ideation is a risk factor for suicide [4] and is associated with mental illness [2]. Thus, suicide ideation may to some extent be seen as a proxy for mental health problems among adolescents. Mental health in turn is highly related to other health concerns and future opportunities [42]. Thus, the shown high rates of suicide ideation are of concern.

There is a lack of studies that have compared and tried to explain the influence of cross-national gender inequality on adolescent health. Looze et al. (2018) showed that higher adolescent life satisfaction is associated with higher levels of national gender equality in Europe and North America. This was explained by a stronger culture of social support in more equal countries [25]. Our results indicate similar associations between better mental health and increased gender equality in the countries analysed here. The settings are quite different between our study and theirs, both regarding cultural and economic circumstances, but it is interesting to consider whether similar explanations regarding a culture of individual social support could apply to our results as well. This is, however, a question for further research.

An interesting finding was that national gender inequality was more strongly related to suicide ideation in boys than in girls, although it was still significant for girls after controlling for selection bias due to school enrolment. Previous studies have indicated that in high-income countries, traditional masculinities can be associated with more suicidal behaviour [43–45]. For example, Coleman [44] studied survey data from college students in the US and found an association between what is often considered traditional masculine behaviour (domineering, being distrustful, self-centred, and competitive) and suicide ideation. Gender theories discuss that traditional masculinities or gender relations may negatively affect suicidality of men in general or subgroups of men [20,21]. Possibly, a higher prevalence and acceptance of traditional masculinities in less gender equal countries and communities could be a reason for the observed differences in the association between gender inequality and suicide ideation. The results of our study could support this, by indicating that gender inequality affects boys’ suicide ideation more than girls’. Hence, one could argue that our study adds to the available evidence that gender equality can be beneficial for the health of both boys and girls, and should be a prioritized issue to increase mental health of adolescents.

The GII is an index of gender inequality, where a higher value represents a greater gender disparity and loss to human development due to these disparities. It is a generic measure that operationalizes gender inequality as the level of female reproductive health (maternal mortality rate and adolescent birth rates) along with gender gaps in empowerment (parliamentary seats and secondary education) and labour market participation. Other factors of relevance for suicide ideation may, for instance be societal norms and attitudes. Further research should clarify whether taking also these aspects of gender relations into account would further strengthen the conclusions here.

The GII index has been criticized for being complicated and favouring countries with better economy [46]. It combines gender gap measurements (such as parliamentary representation and labour participation rates) with absolute numbers of maternal mortality rates and adolescent birth rates [29]. Critics argue that the latter two are highly correlated with GDP/capita, which affects the usefulness of GII. This criticism was however contested by the current study, which found that the GDP/Capita showed no association with suicide ideation while GII did. It could also be argued that both maternal mortality rates and adolescent birth rates [47–49] are important to adolescents’ mental health and future opportunities and are therefore relevant to include as parameters in the current study. High maternal mortality rate will also indicate that more adolescents could be motherless, which may affect the mental health of both boys and girls.

National rates of secondary school enrolment were included in our analyses due to the fact that only adolescents attending school where included in the survey. In high-income countries, this means close to all in the relevant age group. However, in many low- and middle income-countries, the enrolment rate is low in secondary school, and substantially different between the countries included in our study. In such countries, the children in secondary school could be among the privileged in their country and therefore less prone to suicide ideation compared to children who are not in school. One review article from India shows that depression and suicidal thoughts and behaviors are more prevalent among school absentees and school drop-outs [9]. Our results indicate such a selection bias, since higher secondary school enrolment was associated with more suicide ideation, which we interpret to mean that in low-enrolment countries, adolescents with suicide ideation were probably underrepresented. This selection bias might be stronger for girls, since the association was significant for boys even before controlling for school enrolment. School enrolment was also higher in more equal countries, thus creating a selection bias related to both exposure and outcome (in more equal countries, the at-risk adolescents were more likely to be in school and thus included in the sample).

As indicated by the MOR in our analyses, cross-country variation in suicide ideation was relatively high. This was somewhat explained by national gender inequality and then further explained by school enrolment rate (the latter indicating the bias of our sample due to different school enrolment).

There are of course other country-level circumstances that could explain the association found here; or could interact with gender inequality in complex ways that we did not have the statistical power to explore fully. Future research is needed to analyse the role of, among other things, culture, religion, economic inequality, historical context, and norms regarding, for example individuality and secularism.

### Strengths and limitations

This study was one of the first to investigate the current topic cross-nationally. It had a substantial number of participants. Also, by using different sources for the independent and dependent variables, we avoided double reporting bias.

A weakness of the study is that only students enrolled in a school were included, which has been controlled for to some extent by including secondary school enrolment rates. However, school enrolment and gender inequality was quite strongly correlated, which could create problems of multicollinearity.

Another weakness is that the design was cross-sectional, and individuals or countries were not followed over time, making it difficult to state causality. Also, data was collected different years for different countries, which has been adjusted for by including year of survey in the analyses.

With an independent variable at the country level, results could be sensitive to influential outliers or confounders on country level. However, results were relatively robust to excluding outlier countries.

Two of the countries had a considerably larger sample than the rest (Argentina and Malaysia). Since the focus of the analyses was on the second level variables, the sample size in each cluster should not affect the results, but to ascertain this we performed sensitivity analyses which returned the same conclusions.

The Global School-based Student Health Survey is a valuable source of information. It uses the same question regarding suicide ideation worldwide and the material is collected in similar ways. However, cultural aspects such as religion [50], acceptability of suicide [12], norms and trust towards institutions may have affected how the question was interpreted and answered.

## Conclusion

In line with previous findings, the current study indicates that suicide ideation is common among adolescents in the countries studied here, particularly among girls, but also that the prevalence and gender ratios may differ greatly between countries. The current study adds on that a higher national gender equality may be associated with lower rates of suicide ideation among adolescents, especially among boys but also among girls. Among girls and both sexes combined, this association was only significant after controlling for national levels of secondary school enrolment, which indicates that the result may apply to the general population of adolescents, but not to the population of school students.

As suicide ideation among adolescents is an indicator of mental ill health and a strong risk factor for death by suicide, the current results provide support to policy makers and public health scientists in advocating for better gender equality on a national level. This also includes support to public health professionals in focusing on girls’ and women’s reproductive health, rights and opportunities, for the benefit of the whole population.

Our results are also a piece in the puzzle of why suicide ideation differs between countries. Future research should test how the health care sector, NGOs and politicians in low-and middle-income settings can secure a high national gender equality and how this may influence mental health problems and its consequences.

## Data Availability

All data used in this study is publicly available. The GSHS is available for download at CDC’s website [https://www.cdc.gov/GSHS/], all other data sources can be found in the reference list.

## Paper context

Gender equality has been found to affect the mental health of adolescents in high-income countries, but research in low- and middle-income countries is lacking. We found an association between higher levels of national gender equality with lower rates of suicide ideation among boys and girls, in mainly low- and middle-income countries. The association seems to be stronger for boys than girls. This can support policy makers in advocating for increased gender equality.
